# Smokefree Policies in Latin America and the Caribbean: Making Progress 

**DOI:** 10.3390/ijerph9051954

**Published:** 2012-05-21

**Authors:** Ernesto M. Sebrié, Verónica Schoj, Mark J. Travers, Barbara McGaw, Stanton A. Glantz

**Affiliations:** 1 Department of Health Behavior, Roswell Park Cancer Institute, Buffalo, NY 14263, USA; Email: mark.travers@roswellpark.org; 2 InterAmerican Heart Foundation Argentina, Buenos Aires 1425, Argentina; Email: veronica.schoj@ficargentina.org; 3 Heart Foundation of Jamaica, Kingston 5, Jamaica; Email: pmjctc@infochan.com; 4 Center for Tobacco Control Research and Education, Cardiovascular Research Institute, Department of Medicine (Cardiology), University of California, San Francisco, CA 94143, USA; Email: glantz@medicine.ucsf.edu

**Keywords:** Framework Convention on Tobacco Control, secondhand tobacco smoke, public policy, smokefree evaluation, tobacco industry interference, tobacco control legislation

## Abstract

We reviewed the adoption and implementation of smokefree policies in all Latin American and the Caribbean (LAC) countries. Significant progress has been achieved among LAC countries since the WHO Framework Convention on Tobacco Control (FCTC) was adopted in 2005. Both national and sub-national legislation have provided effective mechanisms to increase the fraction of the population protected from secondhand tobacco smoke. Civil society has actively promoted these policies and played a main role in enacting them and monitoring their enforcement. The tobacco industry, while continuing to oppose the approval and regulation of the laws at legislative and executive levels, has gone a step further by litigating against them in the Courts. As in the US and elsewhere, this litigation has failed to stop the legislation.

## 1. Introduction

After years during which the tobacco industry and its allies blocked effective smokefree policies during the 1980s and 1990s, the adoption of the World Health Organization Framework Convention on Tobacco Control (FCTC) in February 2005 changed the policy environment that led to substantial progress in Latin America and the Caribbean (LAC). Implementation of FCTC Article 8 on protection from exposure to secondhand tobacco smoke (SHS) has been achieved in Latin America invigorated and accelerated up the Smokefree Americas Initiative launched by the Pan American Health Organization in 2001. The key to success has been a combination of five factors: professionalized advocacy groups multiplying in the region, a coordinated network of tobacco control advocates and researchers, collaboration between the government and civil society, and technical support provided by international organizations, and available funding to support tobacco control activities in Latin America mainly from developed countries [1].

This review article updates our 2007 paper [2] to April 2012, describing the adoption and implementation of smokefree policies, including its challenges and obstacles. It also discusses the new smokefree initiatives that are starting to emerge among the English-speaking Caribbean countries. Finally, a summary of research studies evaluating smokefree polices conducted in the region is provided.

## 2. Methods

We reviewed smokefree legislation adopted in all LAC countries since 2005, and analyzed the litigation cases against the legislation from two databases maintained by the International Legal Consortium of the Campaign for Tobacco-Free Kids (available at: http://www.tobaccocontrollaws.org/legislation/ and at: http://www.tobaccocontrollaws.org/litigation/). The litigation database is a project in collaboration with the O’Neill Institute for National and Global Health Law at Georgetown University and includes all cases that have reached a country’s Supreme Court. In addition, we collected information on compliance and enforcement from local tobacco control advocates. Finally, we reviewed published peer-reviewed literature available in PubMed related to smokefree policy evaluation conducted in the LAC region.

## 3. Results and Discussion

### 3.1. Smokefree Models

Since 2005, countries in Latin America have been using two different effective strategies to become 100% smokefree: national and sub-national legislation. A 100% smokefree policy means that smoking tobacco is prohibited in all enclosed public places, workplaces (including restaurants and bars) and public transportation, without any exceptions. Meanwhile, as it has in the United States [3,4,5], the tobacco industry continues promoting among policymakers its “accommodation language” to oppose effective laws. This language consists of adding exceptions to legislation such as smoking designated areas, smoking venues exclusive for adults, owners’ voluntary choice whether to become smokefree or not, and/or ventilation and air filtration. 

#### 3.1.1. Effective National Legislation: The Uruguayan Model

In March 2006, Uruguay became the first country in Latin America to adopt a 100% smokefree national policy, which started as a Presidential Decree issued in 2005, and was codified later when the Congress passed a national law in 2008. By April 2012, six countries had followed Uruguay’s example by enacting 100% smokefree legislation (Panama and Colombia in 2008, Peru in 2010, Venezuela and Brazil in 2011, and Costa Rica in 2012) and four approved comprehensive smokefree laws with some exceptions (see 3.1.1.1. Exceptions, below, Table 1). Except Venezuela, that issued a Ministerial Regulation by the Ministry of Health, all of these countries passed national laws. Similar to Uruguay, Colombia first issued a Ministerial Regulation by the Ministry of Social Protection (2008), which was followed a year later by a national law enacted by the Congress (2009). The administrative path (issued by the executive branch) was a strategic mechanism used in some countries to start the process towards adoption of a smokefree law (approved by the national congress), which represents a more permanent legal change than a presidential decree or a ministerial regulation. 

Except for Guatemala and Venezuela, the rest of the countries included the smokefree provisions as part of broader national tobacco control measures. Peru (2010), Brazil (2011), and Costa Rica (2012) passed laws to become 100% smokefree by strengthening existing ineffective national tobacco control laws (from 2006, 1996, and 1995 respectively) which allowed smoking designated areas (SDAs) in bars and restaurants. 

##### 3.1.1.1. Exceptions

Although minor in comparison to the exemptions that the tobacco industry seeks (see 3.1.3. Tobacco industry’s accommodation and ventilation), some exceptions have been slipped in the laws of Guatemala (2009), Honduras (2010), Ecuador (2011), and Argentina (2011). Guatemala and Ecuador laws allow for allocating a percentage of hotel rooms for smoking (up to 20% and 10%, respectively). Honduras law allows for building smoking cubicles within cigars manufactures. The Argentina law allows smoking in private enclosed workplaces without employees working in the same facility and that do not deal directly with the public, and permits the creation of smoking clubs or tobacco stores with the exclusive intention of testing or consuming tobacco products in special areas authorized by proper authorities [6]. Further regulations of the Argentina law were expected to be issued by the Ministry of Health within six months after approval of the law in the Congress, in the hope of clarifying the scope of these exceptions. However, as of April 2012, the regulation of the law had not been issued. 

##### 3.1.1.2. Smokefree Outdoor Areas

In addition to indoor enclosed places, the smokefree legislation includes selected outdoor areas in Uruguay, Panama, Peru, Honduras, Venezuela, Argentina, and Ecuador, health care and educational institutions in Uruguay, Peru, Argentina and Ecuador, sports facilities in Panama, outdoor areas of workplaces in Peru, and areas within 2 meters of public places in Honduras (Table 1). 

**Table 1:** Comparison of comprehensive national smokefree policies in Latin America and the Caribbean (2008–2012).

	Uruguay (2008)	Panama (2008)	Guatemala (2008)	Colombia (2009)	Trinidad & Tobago (2009)	Honduras (2010)	Barbados (2010)	Peru (2010)	Venezuela (2011)	Ecuador (2011)	Argentina (2011)*	Brazil (2011) *	Costa Rica (2012) *
Indoor public places	100% smokefree	100% smokefree	Except 20% hotel rooms	100% smokefree	100% smokefree	100% smokefree	100% smokefree	100% smokefree	100% smokefree	Except 10% hotel rooms	Except smoking clubs and tobacco stores	100% smokefree	100% smokefree
Indoor workplaces	100% smokefree	100% smokefree	100% smokefree	100% smokefree	100% smokefree	Except cigars manufactures	100% smokefree	100% smokefree	100% smokefree	100% smokefree	Except private enclosed workplaces without employees and without services to the public service	100% smokefree	100% smokefree
Public transportation	100% smokefree	100% smokefree	100% smokefree	100% smokefree	100% smokefree	100% smokefree	100% smokefree	100% smokefree	100% smokefree	100% smokefree	100% smokefree	100% smokefree	100% smokefree
Outdoor areas	Health care & educational institutions	Sports facilities	NS	NS	NS	Within 2 meters of public places	NS	Health care & educational institutions	NS	Health care & educational institutions (except University)	Health care & educational institutions (except University)	NS	NS
Sanctions	Warning Fines Temporary closure	Warning Fines Closure	Fines	Warning Fines Suspension of health license	Fines Imprisonment	Fines	Fines Imprisonment	Fines	Fines	Fines	Fines Closure	NS	Fines Closure
Enforcement Agency	Ministry of Public Health	Ministry of Health and federal police	Ministry of Public Health	Health authorities and police	Ministry of Health	IHADFA	Ministry of Health	Ministry of Health	Ministry of Health	Ministry of Public Health	Ministry of Health	NS	Ministry of Health
Other tobacco control measures included in the same legislation	Yes	Yes	No	Yes	Yes	Yes	No	Yes	No	Yes	Yes	Yes	Yes
* Regulation pending, NS: Not specified													

#### 3.1.2. Effective Sub-National Legislation: The Argentinean Model

Similar to United States, Canada and Australia, some countries in LAC implemented smokefree policies at the sub-national level (e.g., province/state or municipalities). These countries in general are characterized by a bigger territory, higher population, and have a federal decentralized political system. In 2005, Argentina became the first LAC country to adopt an effective smokefree policy at the sub-national level [7]. Since then, Venezuela [2], Mexico [8] and Brazil [9,10,11] have been following the Argentinean example. In 2011 Argentina and Brazil passed national laws and Venezuela approved a Ministerial Regulation that applies to the entire country. 

On 30 June 2005, the Province of Santa Fe of Argentina passed 100% smokefree law making Argentina the first country in Latin America to adopt this type of legislation at the sub-national level [7]. Soon after, the provinces of Tucumán (2005) and Neuquén (2007) followed suit. By April 2012, the city of Buenos Aires and eight provinces of Argentina (Córdoba, Mendoza, Entre Ríos, Santiago del Estero, San Luis, Río Negro, Formosa, and Chubut) adopted comprehensive smokefree laws although with some exceptions (e.g., psychiatric hospitals, casinos, jails, smokers’ clubs and gambling establishments) covering a total of around 17 million people, 43% of the population of Argentina. 

In 2006, the State of Monagas (population 900,000) in Venezuela implemented a 100% smokefree law and then the District of Caracas and four cities in the States of Miranda and Nueva Esparta, did the same. 

In 2008, Mexico City passed an amendment to their nonsmokers’ rights law to become the most populous city in the world with a strong smokefree law [8], and the State of Tabasco passed a similar law, which also included smokefree outdoor public places such as parks. In 2011, the States of Morelos and Veracruz followed suit. These sub-national jurisdictions have a total of almost 20 million people that accounts to 18% of the population of Mexico. 

Since 2008, several cities and states in Brazil, started to implement sub-national smokefree policies in accordance with FCTC recommendations. In May 2008, the city of Río de Janeiro (over 6 million people) issued a decree declaring the first 100% smokefree city in Brazil. In October 2008, the State of Rondonia passed the first 100% smokefree state law in Brazil. The same was achieved in 2009 in the States of Sao Paulo, Rio de Janeiro, Parana, Amazonas, Roraima, and Paraiba [12]. These seven sub-national jurisdictions have a population of about 80 million people that accounts to 42% of the population of Brazil that as of April 2012 were protected from SHS exposure.

#### 3.1.3. Tobacco Industry’s Accommodation and Ventilation: The Spanish Model

In 2006, Chile passed an ineffective national tobacco control law that nominally regulated smoking in public places. Rather than providing effective protection from SHS, the law allowed for SDAs in most public places, replicating the weak 2005 Spanish law in Latin America, which allowed for SDAs in bars and restaurants [13,14], mirroring the tobacco industry’s “accommodation” (Courtesy of Choice) program [4,5]. The Spanish law was amended in 2010 to become 100% smokefree (except in up to 30% of hotel rooms); as of April 2012, Chile had not strengthened its law.

##### 3.1.3.1. Other National Laws that Do not Comply with the FCTC

In addition to Chile, Peru (2006, later changed), Bolivia (2007), Mexico (2008), Nicaragua (2010), and El Salvador (2011), adopted ineffective national legislation that follows tobacco industry policies designed to minimize impact on smoking. These laws are characterized by the inclusion of one or more of the following exceptions: SDAs (completed isolated or not), use of ventilation or air filtration systems, smoking venues exclusively for adults, owners’ authority to voluntary choose whether to become smokefree or not. These laws are not in accordance with WHO recommendations [15,16] nor WHO FCTC Article 8 Guidelines [16].

##### 3.1.3.2. Sub-National Laws that Do not Comply with the FCTC

Similarly, the tobacco industry has also been successful in blocking effective smokefree policies at the sub-national level. In 2008, the Province of Buenos Aires in Argentina passed a law that allowed SDAs in most of the venues after a strong lobby of the industry in the provincial congress [17]. A similar situation happened in 2009 in the Province of La Pampa. Several jurisdictions enacted ineffective legislation allowing for SDAs in Brazil (Espirito Santo, Minas Gerais, Rio Grande do Sul, Santa Catarina, Goias, Mato Grosso, Mato Grosso do Sul, Para, Tocantins, Bahia, Ceara, Maranhao, Sergipe, Pernambuco, Alagoas, and the Federal Distric) [12] and in Mexico (Aguscalientes, Baja California, Campeche, Chiapas, Chihuahua, Coahuila, Colima, Durango, Guanajuato, Guerrero, Jalisco, Hidalgo, Mexican State, Michoacán, Nuevo León, Oaxaca, Puebla, Quintana Roo, San Luis Potosí, Sinaloa, Sonora, Tamaulipas, Tlaxcala, and Zacatecas) [18]. 

### 3.2. New Challenges and Obstacles

#### 3.2.1. Implementation, Compliance and Enforcement

As of April 2012 one of the main challenges for the region remained achieving successful full implementation of the smokefree legislation. According to a civil society qualitative report [19] that collected information from key informants mostly tobacco control advocates in most of the LAC countries, the level of compliance in the region is heterogeneous. Non-governmental organizations from countries such as Uruguay and Panama have reported a very high level of compliance and great social acceptance of the policy. However, in other countries, local advocates have reported difficulties in the implementation of the legislation, mostly because of lack of monitoring and surveillance by the public officials. In Guatemala for example, it has been reported the lack of a telephone line to report violations of the law or a specific government body to follow-up on such complaints. Low compliance with the law was observed in bars and pubs due to the lack of inspections at night-time. In Colombia, despite lack of political will to strengthen the implementation of the law (there are no national programs or campaigns to raise awareness and to promote the compliance of the policy), a great social acceptance has been reported as well. The same civil society report [19] found that regarding compliance of sub-national legislation, Brazil has reported a high level of compliance in Sao Paulo (99.8% of 361,077 venues surveyed in one year), and a high level of support (97% non-smokers, 92% smokers), and in Rio de Janeiro, 99.3% of the venues supervised complied with the legislation three months after entry into force of the 100% smokefree legislation [19]. On the other hand, the civil society in Mexico pointed out that, despite the high level of compliance in Mexico City, no appropriate sanctions or penalties have been applied in cases of violations to the legislation [8,19].

Quantitative data from population-based representative samples from the International Tobacco Control Policy Evaluation Project (ITC Project) have shown that in 2006 Uruguayan smokers were more likely than Mexican smokers to stronger support for 100% smokefree policies in enclosed workplaces, restaurants, and bars, indicating that comprehensive smokefree policies (such as the one implemented in Uruguay) are likely to increase the social acceptability of smokefree policies implementation [20]. Another study conducted in Mexico comparing reductions in self-reported SHS exposure across key venues (*i.e.*, workplaces, restaurants and cafes, and bars and discos) in Mexico City *versus* other Mexican cities found that social support for ending smoking in those venues increased overall, but at a greater rate in Mexico City than in other cities. Also, self-reported SHS exposure in bars and restaurants had significantly greater decreases in Mexico City than in the rest of the cities, indicating that the comprehensive smokefree law in Mexico City was generally accompanied by a greater rate of change [21]. A third study that compared SHS exposure before and 4 and 8 months after the implementation of the Mexico City law found a high and increasing support for the 100% smokefree policy, although the support did not increase for smokefree bars and that SHS exposure decreased generally but compliance was incomplete, especially in bars [22]. 

#### 3.2.2. Tobacco Industry Interference

Tobacco companies’ opposition to effective smokefree policies continues to be the primary obstacle to progress. As in the US and other countries with a longer history of enacting and implementing strong smokefree legislation and policies, industry strategies include lobbying the executive authorities blocking regulations of the law and even promoting a presidential veto, litigation in the courts, as well as amendments and preemption in the congress after the law passes.

##### 3.2.2.1. Presidential Veto, Blocking of the Regulation, and Amendment of the Law

On 23 June 2011, the Congress of El Salvador passed a national tobacco control law that included 100% smokefree policies. On 18 July, almost a month after the approval, the President of the country vetoed the law. As part of his arguments to support the veto the president repeated well-established tobacco industry claims in the media that “individual freedom is diminished, (the law) also harms economic freedom of the stakeholders that participate in the market, negatively affecting not just the tobacco industry” [23] (this veto resembled the 1992 presidential veto of the Argentina tobacco control law almost 20 years later promoted by behind-the-scenes tobacco industry lobbying [24,25]). Tobacco control advocates with support from international organizations that work in LAC, successfully pressured the legislators to overturn the veto, which the Congress of El Salvador did on 23 July. However, the President refused to issue the regulations of the law and then introduced an amendment in Congress allowing for SDAs. Surprisingly, on November 17, the same legislators that had overridden the veto to keep the 100% smokefree law, changed their position and approved the amendment, a huge success for the tobacco industry [26].

##### 3.2.2.2. Delay of Regulations of the Law

Congresses of Argentina and Brazil passed national smokefree laws in 2011. However, the executive authorities of Argentina have not issued the regulation necessary to implement the law and local advocates have claimed this delay could have been as a result of tobacco industry interference. Furthermore, it was not clear how the regulation of the law would solve the potential loopholes of the Argentina law. The Brazilian law did not specify any timeline for issuing the regulation and therefore its implementation was pending as of April 2012. 

##### 3.2.2.3. Litigation

As previously in the US [3], since the enactment of effective smokefree policies at the national and sub-national levels, the tobacco industry has began litigation process in several countries of the region: Argentina, Mexico, Brazil, Guatemala, Peru, and Paraguay (Table 2). 

**Table 2:** Main litigation cases against smokefree legislation in Latin America (2006–2012).

Country (year)	Issues & legal arguments	Plaintiff	Defendant	Outcome
Argentina (2006)	Unconstitutionality of Law 1.799 of Buenos Aires City; significant decrease in sales, right to licit industry, smokers’ discrimination, freedom of intimacy, principle of reasonability	30 injunctions from owners of bars, cafeterias, restaurants, bingos, and mall.	City of Buenos Aires	Rejected
	Unconstitutionality of Laws 12.432 & 12.605 of Province of Santa Fe and Ordinance 8021of Rosario City	Owner of a cafeteria	Municipality of Rosario & Province of Santa Fe	Rejected
	Unconstitutionality of Ordinances 11039 and 11040 of Córdoba City	Bars and restaurants Association of Cordoba	City of Córdoba	Rejected
	Unconstitutionality of Ordinance 10.866 of Neuquén City	3 injunctions from casino, bingo	City of Neuquén	2 rejected, 1 pending
Argentina (2008)	Unconstitutionality of Law 12.432	BAT	Province of Santa Fe	Pending
Mexico (2009)	Unconstitutionality of Mexico City’s protection for the health of nonsmokers law	1,000 injunctions Tobacco industry	Mexico City	The Supreme Court ruled in favor of maintaining the law as it is
Guatemala (2009)	Violation of the freedom of industry and commerce	Guatemala Chamber of Commerce	Guatemala Republic	The Supreme Court ruled in favor of upholding the law to protect the right to health
Brazil (2010)	Unconstitutionality of Smokefree Sao Paulo law; exceeded its powers in the matter	Bars and Restaurants Association of Sao Paulo (ABRASEL)	Consumer Defense and Protection of Sao Paulo (PRECON)	The Higher Court upheld the Sao Paulo law
Paraguay (2010)	Unconstitutionality of the Presidential Decree 4.174, executive branch had no authority to regulate FCTC implementation	Tobacco industry	Executive branch of Paraguay	The Supreme Court ruled against the Presidential Decree arguing that this type of regulations needed to be adopted by the Congress.
Peru (2011)	Unconstitutionality of Article 3 of Law 28.705; infringement of the right to personal autonomy, right to commerce, and right to economic freedom	5,000 Peruvian citizens	Peru Republic	The Supreme Court ruled in favor of upholding the law
Costa Rica (2012)	Unconstitutionality of the 2012 tobacco control law	10 legislators of the Congress of Costa Rica	Costa Rica	The Supreme Court ruled in favor of upholding the law

In Argentina, between September and November, 2006, 30 injunctions were filed before the local Court in the City of Buenos Aires arguing the unconstitutionality of tobacco control law 1.799. All were rejected. Similar situations experienced the sub-national laws from Mexico and Brazil. 

Legal challenges to the national laws in Guatemala and Peru reached the Supreme Courts of these countries where actions of unconstitutionality were filed. Both Courts ruled that the laws were constitutional and that the right of health was more important than the commercial right. 

In 7 April 2010, the President of Paraguay issued Presidential Decree No. 4174 declaring the entire country 100% smokefree. Soon after, the tobacco industry filed a lawsuit to suspend the decree, arguing that it was unconstitutional. In November, 2010 the Supreme Court ruled in favor of the tobacco industry, stating that only the congress, not the executive branch, had the authority to implement the FCTC. Despite the success of the tobacco industry, a jurisdictional ruling like in Paraguay, is much narrower than a rule on underlying rights claims. After litigating against the decree, the tobacco industry through allies in the Senate strongly lobbied the Congress to pass an ineffective national law, which allowed SDAs in most of the public venues and the voluntary regulation by owners. Legislators discussed the bill and, despite opposition of civil society, the law passed. Local tobacco control advocates along with allies in the Ministry of Health and support from regional advocates from Argentina and Uruguay, pressured the President to veto the law. Finally the law was vetoed; a success for public health advocates [19]. As of April 2012, Paraguay had not approved any other smokefree initiative at the national level. 

On 27 February 2012, the Congress of Costa Rica passed a tobacco control law that included the adoption of 100% smokefree policies, modifying a previous ineffective 1995 law [27]. Despite strong efforts by the Red Nacional Antitabaco Costa Rica (RENATA, the Costa Rican anti-tobacco network) to help pass the bill, soon after and while the bill was awaited for the President’s signature to become law, 10 legislators filed a petition to the Supreme Court challenging the law. The Court suspended the bill [28] until March 20 when it upheld the law by ruling that it was constitutional. 

##### 3.2.2.4. Preemption

In 2008, almost immediately after Mexico City enacted a 100% smokefree law, the national Mexican Congress approved a federal law, which allowed for SDAs. Pro-tobacco interests then challenged the Mexico City law in Court, claiming that the federal law preempted it. The Supreme Court upheld the Mexico City law, ruling that a sub-national jurisdiction has the authority to go beyond the federal law to protect the fundamental right of health to all citizens [8]. A similar attempt might be possible in other countries like Argentina where a national law approved in 2011 would allow for some exceptions in a country that have already implemented 100% smokefree provincial laws [7]. 

### 3.3. New Initiatives

As of April 2012, smokefree initiatives had been introduced or were being discussed in the Congress in Chile and Mexico to amend their current national laws and become 100% smokefree. In September, 2011 a bill was introduced in the congress to modify the 2006 law to make Chile 100% smokefree. This initiative has been strongly promoted by the civil society under the coalition Chile Libre de Tabaco (Smokefree Chile). Similarly, an amendment of the 2008 Mexican law was being supported by the civil society including Fundación InterAmerican del Corazón Mexico (InterAmerican Heart Foundation Mexico) and others. On 31 May 2011, at a press conference celebrating the World No Tobacco Day [29,30], the Minister of Health and Sports of Bolivia announced that the country would modify its 2007 law, which allows SDAs and smoking venues, to become 100% smokefree. However, as of April 2012, no action had been taken in Bolivia.

### 3.4. Is the Caribbean Ready to Follow?

Caribbean countries face two different realities. While smokefree initiatives are emerging among the non-Latin Caribbean countries (English and Dutch speaking), the Latin Caribbean (Spanish and French speaking) is lagging behind. 

In December 2009, Trinidad and Tobago became the first Caribbean country to pass 100% smokefree law. Satisfactory implementation has been reported in Port-of-Spain, the capital city and in other cities; however, this is not the case in rural areas where there are no qualified staff responsible for fulfilling compliance surveillance and control [19]. On 1 October 2010, Barbados approved and enacted legislation to end smoking in public places despite tobacco industry opposition arguing that the tourism would be affected. As of April 2012, there were new initiatives under discussion in Jamaica, Guyana, Saint Lucia, and Guyana. 

Jamaica has made little progress in tobacco control legislation. Despite having draft legislation since 2005, there has been a very slow pace of review and drafting of a final bill. In October 2009, a smokefree regulation was submitted to the Chief Parliamentary Council for review, with a view to speeding up the passage of the smokefree portion of the bill. There have been several assurances by the Minister of Health that the bill was forthcoming- but no action [19]. 

As of April 2012, Latin Caribbean Cuba, Haiti and Dominican Republic remained part of the small group of countries that have not even ratified the FCTC. There has been very little or no progress in those countries related to smokefree policy implementation. 

### 3.5. Local Research Studies on Smokefree Evaluation

Along with the dramatic increase in smokefree policy activity in LAC countries, there is a corresponding increase in related scientific research. These studies in the region serve two purposes: to demonstrate the need and desire locally for smokefree policies and to evaluate the impact of these policies after implementation. These studies focus on major smokefree policy research domains [31,32] including public support, compliance, exposure monitoring, health impact, and economic impact. Local research studies demonstrating the need for and evaluating the impact of smokefree policies in LAC had been rare until the last decade. The recent studies have proven to be helpful to promote smokefree policies in other jurisdictions within the region, to protect effective policies from the continuing threats of the tobacco industry, and to demonstrate the ineffectiveness of certain policies. Published studies of SHS-particulate exposure have been done in Argentina, Brazil, Mexico, Panama, Uruguay, and Venezuela and have shown about a 5-fold increase in fine particle pollution levels in places with smoking present compared to those without smoking [33,34,35]. Levels in places with smoking far exceed fine particle health standards set by the Mexican Índice Metropolitano de la Calidad del Aire (IMECA), WHO, and US Environmental Protection Agency. Studies of airborne nicotine concentrations in public places have been published for at least 11 LAC countries and have shown significant nicotine concentrations in a wide range of locations where smoking occurs, including schools, hospitals, government buildings, airports, restaurants, and bars [35,36,37,38]. Research has also shown that implementation of smokefree laws is followed by dramatic reductions in this indoor air pollution. There was an overall 91% reduction in airborne nicotine concentrations in Uruguay public places and worksites after this country’s smokefree air law was enacted [37]. One study of carbon monoxide showed significant decreases in CO concentrations in ambient air and exhaled breath of both smoking and non-smoking workers after Sao Paolo, Brazil’s smoke-free air law was implemented [10]. Studies have shown high levels of public support for smokefree air policies that increase after policy implementation [20,21,22,39] and that media campaigns enhance smokefree policy implementation in sub-national jurisdictions in Mexico [21,40,41] and Brazil [9]. Contrary to tobacco industry claims and consistent with findings elsewhere in the world [42,43], an economic impact found demonstrated no impact of the Mexico City smokefree law on hospitality business revenues [44]. Three studies have assessed health impact of smokefree legislation. The first, conducted in Argentina, showed that the 100% smokefree Santa Fe law was more effective than the Buenos Aires partial smoking restrictions in reducing acute coronary syndrome hospital admissions [45]. The second, conducted in Uruguay, demonstrated a 22% reduction in acute myocardial infarction hospital admissions after 2 years of implementation of the 100% smokefree Uruguay national law [46]. The third, conducted in Neuquén, Argentina, showed a significant decrease in respiratory symptoms in bar and restaurant workers after implementation of a provincial smokefree law [47]. 

Cohort surveys studying all domains of tobacco control policy impact are on-going in Mexico, Uruguay and Brazil as part of the ITC Project (http://www.itcproject.org/countries). Finally, the WHO/CDC Global Adult Tobacco Survey (GATS) conducted in 2008–2009 showed that the percentage of adults exposed to SHS at the workplace was 16.5% in Uruguay [48], 19.7% in Mexico [49] and 24.4% in Brazil [50].

The research being conducted by various local, national, regional, and global organizations that represent civil society, government, and academia on smokefree policy issues bodes well for the future of effective smokefree policies in the LAC region [51]. Quality scientific evidence that is locally relevant is essential to push forward and implement effective policies and is something that was lacking in the LAC region until recently. 

## 4. Conclusions

Significant progress in the implementation of effective smokefree policies has been achieved among LAC countries since the adoption of the WHO FCTC in 2005, even in a country like Argentina that has not yet ratified the treaty. As of April 2012 thirteen LAC countries (out of 33) have adopted a comprehensive national smokefree policy, accounting for almost 67% of the total population in the region (582.6 million [52]). National legislation was still pending in several countries, mostly from the English-speaking Caribbean. Legislation at the sub-national level has provided a very effective mechanism, as an alternative to national legislation, to increase the fraction of the population protected from exposure to SHS. 

Another effective strategy has been the approval of smokefree policies using the executive or administrative path (e.g., presidential decree or ministerial regulation) as a way to start the process towards the adoption of a smokefree law, which has a higher hierarchy. This process was replicated in some countries of the region where after the administrative path, a national law was approved in the Congress such as in the cases of Uruguay, Panama and Colombia. One of the advantages that this path offers is that given a delay of national congress to approve laws, in part as a consequence of tobacco industry interference, the political commitment from the executive branch allows to promptly guarantee the protection of health through the implementation of the measure. In addition, it facilitates establishing the issue in the political and public agendas that allows it to advance in the process of the approval of a law by the congress. A disadvantage of decrees and ministerial regulations is that they can be easily revocable.

The tobacco industry continues to be the main challenge. As more countries adopt and implement effective smokefree laws, the industry’s strategy seems to be shifting from interference at the legislative level to working to delay or undermine executive action to implement these policies as well as pursuing litigation against these laws through constitutional challenges. Both governments and civil society are defending the laws with strong arguments highlighting the rights to health, to life, to clean environment, of the child, and human rights guaranteed by their own constitutions and/or by international treaties (e.g., International Covenant on Economic, Social, and Cultural Rights; Convention on the Rights of the Child; American Convention on Human Rights) [53]. To which they are parties. While the industry has had some success in undermining implementation of smokefree laws, as in the United States [3] their constitutional challenges based on “rights” claims have failed (They had one success on jurisdictional issues.). 

Civil society has actively participated in the promotion of these policies and played a central role in securing enactment and monitoring the enforcement of legislation that complies with the minimum standards of the FCTC and its Article 8 Guidelines. Governments increasingly understand the importance of the adoption of this policy not only to protect the health of their citizens but also to prevent the economic burden of the morbidity and mortality associated to smoking and secondhand smoke. 
